# Menstrual attitudes in adult women: A cross-sectional study on the association with menstruation factors, contraceptive use, genital self-image, and sexual openness

**DOI:** 10.1177/17455057241249553

**Published:** 2024-04-29

**Authors:** Ingela Lundin Kvalem, Ida Maria Dahr Nygaard, Bente Træen, Anna Ivanova, Camilla Lindvall Dahlgren

**Affiliations:** 1 Department of Psychology, University of Oslo, Oslo, Norway; 2 Museum of Cultural History, University of Oslo, Oslo, Norway; 3 Institute of Psychology, Oslo New University College, Oslo, Norway

**Keywords:** contraceptive method, genital self-image, menstrual attitudes, sexual openness, women

## Abstract

**Background:**

Menstruation is a central part of the everyday life of most women, and menstrual attitudes may impact health and well-being.

**Objectives:**

This article aimed to map menstrual attitudes among adult women and examine factors associated with these attitudes, such as aspects of menarche and current menstruation, and rarely studied factors, such as genital self-image and sexual openness.

**Study Design:**

A cross-sectional online survey.

**Method:**

A sample of 1470 women, aged 18–50 years, were recruited through social media sites. The *Menstrual Self-Evaluation Scale* was used to measure three different attitudes: menstruation as natural, shameful, and bothersome. Multiple linear regression analysis was used to investigate the relationship between each attitude and factors related to menarche and current menstruation, contraceptive use, genital self-image (assessed by *Female Genital Self-Image Scale*), and sexual openness (*Personal Comfort with Sexuality Scale*). Sociodemographic variables were included into the models as covariates.

**Results:**

Agreeing with the attitude of menstruation as something natural was predicted primarily by positive emotions at menarche, experiencing less menstrual pain, using no or nonhormonal contraception, and having a positive genital self-image. Perceiving menstruation as bothersome was predicted by a lower educational level, experiencing stronger menstrual pain, having more perimenstrual psychological symptoms, and using hormonal contraceptives. Menstruation as something shameful was chiefly predicted by lower sexual openness and a negative genital self-image.

**Conclusion:**

Many women held attitudes about menstruation as both something natural and bothersome. Menarche and current menstruation experiences, and contraceptive method, played central roles in shaping attitudes toward menstruation as natural and bothersome. Viewing menstruation as shameful stood out from other attitudes by indicating a triad of self-objectified shame that includes menstruation, sexuality, and genital self-image. Further research into the relationships between menstruation, contraceptive use, sexuality, and body image is needed to enhance our understanding of women’s menstrual health.

## Introduction

Despite increased openness and acceptance toward menstruation and women’s health in the public discourse,^1,2^ there are still taboos and stigmas associated with menstruation.^3–5^ Menstruation is often constructed and presented in popular culture and marketing as something bothersome, impractical, nasty, and shameful but also as unnecessary.^6–8^ Simultaneously, menstruating women, and especially premenstrual women, are portrayed as emotional, erratic, aggressive, and out of control.^9,10^ Research has shown that these types of negative attitudes toward menstruation in general and toward menstruating women in particular have consequences for several aspects of women’s physical, mental, and sexual health and may impact health behavior.^11–15^ The fact that girls and women are exposed to attitudes that may have consequences for their health and well-being makes understanding the complex underpinnings of menstrual attitudes a central topic in public health promotion. The focus of this article is to map menstrual attitudes among Norwegian adult women and examine factors that have rarely been studied previously but may be associated with menstrual attitudes, such as contraceptive use, genital self-image, and sexual openness.

Like attitudes in general, menstrual attitudes are believed to be a multidimensional social construct. Despite the similarities in the physiological experience of menstruation for most women worldwide, the perceived experiences may differ considerably due to the differing sociocultural frameworks of understanding or socialization processes.^16^ Attitudes are a product of knowledge, perceptions, emotions, experiences, and behaviors,^17^ and women’s menstrual attitudes can thus be described as a combination of sociocultural, biological, and psychological factors, and personal experiences.

Research on menstrual attitudes has found that there is a widespread sociocultural attitude about menstruation as “disgusting, yet natural”^7^ and that women can have both positive and negative feelings directed toward menstruation simultaneously.^14,15^ In studies with American, Swedish, and Icelandic women, this ambivalence was expressed in the attitude that menstruation is both a sign of good health and an important part of being a woman, and being a burden associated with unpleasant symptoms.^3,15,18^

Roberts^14^ argued that menstrual attitudes are based on more than the approval of cultural beliefs, positive and negative meanings of menstruation, or the effects of menstruation on women. In societies that sexually objectify the female body, the body is largely assessed according to ideals related to the appearance and beauty rather than the biological function.^19^ Since menstruation breaks with beauty ideals related to hygiene, cleanliness, and smell, it may create shame, and shame should thus be added as a central aspect of menstrual feelings and self-evaluations.^14^ In support of this claim, studies have showed associations between a higher degree of self-objectification in women and negative menstrual attitudes, and a sense of global shame about reproductive events, such as menstruation.^10,20^

There are several factors that may be associated with menstrual attitudes in adult women who have rarely been examined before, such as factors related to menarche, current periods, and contraceptive use, and attitudes toward sexuality and genital self-image (i.e. subjective thoughts and feelings about one’s own genitals).^21^

### Menarche

Knowledge about menstruation and the menarcheal experience lays a decisive foundation for women’s menstrual attitudes in adulthood.^22–24^ White^25^ reasoned that young girls with insufficient knowledge may end up passing on misinformation and myths about menstruation to peers and, in the future, to their own daughters. Cultural beliefs about menstruation being shameful, “dirty,” and something one must hide and keep secret, in combination with a lack of knowledge, have proven to be particularly important in connection with girls’ first menstruation.^5,24,26^ For example, research shows that girls frequently report negative emotions at menarche, such as shame, disgust, fear, and anger, which may prevail and influence adult menstrual attitudes.^24,27^

### Current menstruation and contraceptive use

Women who experience heavy and irregular menstrual bleeding, unpleasant symptoms, and pain tend to report more negative attitudes than women with lighter, less painful and more regular periods.^28^ Despite the fact that several methods of contraception may affect menstruation regularity, duration, amount of bleeding, and pain, and therefore can be assumed to influence menstrual attitudes, studies exploring the link between contraceptive methods and menstrual attitudes are still scarce. One example is a study by Morrison et al.,^29^ which found that hormonal contraceptive users were less likely to view menstruation as natural. Findings from research on women’s attitudes toward hormonal contraception and menstrual suppression may also give some indications.^8,30^ Hormonal contraception, which was originally developed to prevent pregnancy, has increasingly been used to control menstruation and the menstrual cycle.^15^ The finding that it is medically unnecessary for women to bleed when using hormonal contraception has been marketed as a way to escape menstruation completely and “freeing” women from this biological function, portraying menstruation as an unnecessary obstacle to modern women’s success and careers.^6,31^ For example, Chrisler et al.^11^ showed that women’s tendency for self-objectification to distance their “self” from their body was associated with more positive attitudes toward suppressing the menstrual cycle.

### Sexual openness and genital self-image

Several researchers have pointed out how women’s sexuality and menstruation are linked to the same bodily region, namely, the genital area,^13,15,32^ which may impact menstrual attitudes. This link may create a psychological bridge between sexuality, menstruation, and genital self-image, and negative or positive thoughts and feelings related to one of these aspects can thus easily spread over to the other two. In line with this, researchers have posited that young girls who develop negative attitudes and reactions to themselves as sexual beings or to their bodies as sexual instruments may also develop negative attitudes toward menstruation, and vice versa.^13^ In a study of adult women, Schooler et al.^15^ found that women with more shame associated with menstruation reported fewer sexual experiences, more sexual risk behaviors, being more uncomfortable getting involved in sexual situations, and found it more difficult to adhere to their own sexual needs and boundaries. Women with positive attitudes toward menstruation reported instead being more comfortable with their own sexuality and having a more open view of sexuality in general.^13,33^

Although studies have found an association between menstruation and related topics, such as sexuality and general body image,^11,13^ to our knowledge, no quantitative studies have explored the association between genital self-image and menstrual attitudes. It is, however, reasonable to assume that genital self-image will be related to menstrual attitudes in a similar way as general body image. For example, several studies have found that general body shame is closely related to menstrual shame.^12,14,20^ Several qualitative studies that have examined women’s thoughts and feelings related to their genital area found that some women considered the genital area unappealing because it is where menstrual bleeding comes from^34^ or described it as a source of frustration, as dirty and disgusting, and in need of control and maintenance.^35,36^ In line with self-objectification theory, DeMaria et al.^35^ noted that women’s genital self-image often limited their daily activities and sexual experiences due to emotions, such as self-consciousness and fear of being judged.

### The current study

The aim of this study was to map menstrual attitudes among Norwegian adult women and to investigate how variables related to first and current menstruation, method of contraception, sexual openness, and genital self-image were related to and predicted different menstrual attitudes.

## Methods

### Sample and procedure

The study data stem from a web-based cross-sectional survey of menstrual attitudes in a convenience sample of Norwegian women. The data collection took place from September to October 2021. The survey was administered by the Department of Psychology, University of Oslo. The study was promoted through two private Facebook groups exclusive to female members: “Kvinnesnakk” (“Women’s Talk”; 6400 members) and “Slay Squad: Older Generation” (3500 members). In addition, an email was distributed to “Femihelse” (“Femhealth”), a podcast focusing on women’s health. The podcast shared the survey link through an Instagram “story” in a video that encouraged followers to participate.

The promotional materials included a link redirecting participants to a website containing information about the survey and providing access to the online questionnaire. The heading of the project information page was “Do you want to participate in the research project “Menstruation and health”?,” followed by the aim of the study, which was “to investigate the association between menstrual attitudes and genital self-esteem in Norwegian women, and to explore whether this association relates to sexual satisfaction, and how comfortable and open one is in relation to one’s own sexuality.” A total of 1927 people clicked on the information page, and 1500 consented to participate and were sent on to the questionnaire. Two inclusion criteria questions regarding gender (woman) and age (18–50 years) were employed as entry points to the questionnaire. Overall, 28 participants were excluded from the study as they did not meet both main criteria. In addition, two participants were excluded due to self-reporting pregnancy. The final study sample consisted of a total of 1470 female participants between 18 and 50 years of age.

To determine the required size for this web-based convenience sample, we used a sample size calculator suitable for random sampling. The calculation indicated that a sample of 670 participants was necessary to achieve a 99% confidence level, ensuring that the true value falls within a margin of ± 5% of the value obtained from the survey.

### Measures

#### Menstrual attitudes

To capture the central aspect of shame in menstrual experiences, attitudes toward menstruation were assessed using *the Menstrual Self-Evaluation Scale*.^14^ This 16-item scale, adapted^[Bibr bibr14-17455057241249553]^ from the *Menstrual Attitudes Questionnaire* (MAQ), developed by Brooks-Gunn and Ruble,^37^ encompasses two of the subscales (10 items) from the MAQ.

The first subscale, originally named “Menstruation as a Natural Event,” was split into two separate factors in Robert’s modified scale: “Menstruation as Enabling Awareness of One’s Body” and “Menstruation as Life Affirming” (example item “Menstruation provides a way for me to keep in touch with my body”). The second subscale, “Menstruation as a Bothersome Event,” includes statements such as “I hope it will be possible someday to get a menstrual period over within a few minutes.”

Furthermore, an additional factor comprising six items was introduced in the work by Roberts^14^ to capture more negative and shameful feelings related to menstruation (e.g. “I would prefer not to talk openly about menstruation”). Participants rated the items on a seven-point Likert-type scale ranging from *strongly disagree* (1) to *strongly agree* (7).

Due to the initial factor’s low internal consistency (Cronbach’s α = .59) on the “Menstruation as a Bothersome Event,” a new exploratory factor analysis was conducted for the study sample (refer to Supplement Table). This analysis yielded a slightly different solution than Roberts’s findings. In our study, the factors “Menstruation as Enabling Awareness of One’s Body” and “Menstruation as Life Affirming” emerged as a single factor. In addition, Items 1 and 2, which originally loaded within the “Menstruation as a Bothersome Event” factor in both the MAQ^37^ and Roberts’s^14^ version, loaded together with items from the original MAQ factor “Menstruation as a Natural Event” in this study sample. The three resulting factors in the current study were labeled as follows: “Menstruation as something natural,” “Menstruation as something shameful,” and “Menstruation as something bothersome,” with corresponding Cronbach’s alpha values of .85, .78, and .70, respectively. A high score on each subscale indicated a strong agreement with the attitude expressed in the title, reflecting the participant’s perspective on menstruation.

#### Current menstruation

Participants were asked about different aspects of their menstruation. *Frequency of periods* was measured with the question “How often do you have your period/are you bleeding? (If you are on hormonal contraception that has changed or removed your period, please answer based on your current bleeding pattern),” and the response categories ranged from *regularly once a month* (1) to *never* (6), with the option *other, please specify*. When applicable, responses provided under the “Other” category were incorporated into the existing categories. For instance, responses such as “Breastfeeding, so no period now” or “Never, Have PCOS, Have not had a period for over 4 years” were included under the “Never” category. In addition, due to a notable number of responses under “Other” indicating a more frequent bleeding pattern than once a month, a new category was introduced, and existing response categories were reorganized as follows: (1) *twice or more monthly*, (2) *regularly once a month*, (3) *regularly/irregularly every 2/3 months*, (4) *once or twice a year*, and (5) *never. The length of the period* was estimated with the question “How many days do you usually bleed when you have your period?” with response options of (1) *1–3 days*, (2) *4–5 days*, and (3) *6 days or more. Amount of bleeding* and *menstrual pain* were assessed by asking the question: “On a scale from 1 to 7, where 1 is “nothing” and 7 is “very much,” please answer the following: How much do you tend to bleed when you have your period?/ How much pain do you usually experience in connection with your menstruation?” *Perimenstrual psychological symptoms* were measured with a follow-up question after the question about pain: “To what extent do you currently experience other physical or psychological menstruation-related symptoms before or when you have your period?” followed by four physical and four common psychological symptoms with response categories ranging from *not at all* (1) to *very much* (5). In this study, only the averages of the four psychological symptoms, namely “depression/sadness,” “irritation,” “anger/hostility,” and “anxiety,” were incorporated. The internal consistency of this composite variable was Cronbach’s α = .84.

#### Contraception

Contraceptive method was assessed by the question “Do you use any form of contraception? (If you use hormonal contraception in combination with a condom or other, indicate which hormonal contraception you use),” with the response options: (1) *long-term hormonal contraception (contraceptive implant, hormonal intrauterine device (IUD))*, (2) *other hormonal contraception, (contraceptive pills, mini-pills, contraceptive ring/patch/injection)*, (3) *hormone-free contraception, (condom, diaphragm, nonhormonal IUD, fertility awareness methods (FAM), apps)*, and (4) *do not use contraception*. Based on these options, a dichotomous variable with *nonhormonal/no contraceptive* versus *hormonal contraceptive use* was constructed.

#### Menarche

*Age* at first period was asked as an open-ended question. *Knowledge* about menstruation at menarche was measured with the question “How much knowledge about menstruation did you have when you got your period for the first time?,” with response options ranging from *no knowledge* (1) to *very much knowledge* (5). *Emotions* at menarche were assessed with the question “To what extent did you experience the emotions mentioned below when you had your first menstruation?” followed by nine emotions rated on a scale from *to a very small extent* (1) to *a very large extent* (5). The four positive emotions (proud, happy, mature, and excited) were averaged and called “proud and happy,” while “concerned and afraid” consisted of the five emotions with negative meaning (shameful, afraid, sad, worried, and weird), with Cronbach’s alpha values of .85 and .88, respectively.

***Sexual openness*** was measured with the *Personal Comfort with Sexuality Scale*^13^ (α = .81 in the current study). The scale consists of 10 statements that assess the degree of feeling comfortable and open with and accepting of sexuality, privately and publicly (e.g. “I think I’m a sexy person” and “I feel comfortable talking about sexuality with strangers”). The response categories ranged from *strongly disagree* (1) to *strongly agree* (7), with a high mean score indicating more sexual openness.

***Genital self-image*** was measured using the *Female Genital Self Image Scale*^38^ (α = .85). The scale included seven statements related to women’s feelings and thoughts about their own genitals (e.g. “I am satisfied with the appearance of my genitals” and “I am not embarrassed about my genitals”). Agreement was indicated from *strongly disagree* (1) to *strongly agree* (4), with a high mean score indicating a positive genital self-image.

#### Sociodemographic variables

The age of the participants was indicated by six age groups: 18–24, 25–29, 30–34, 35–39, 40–44, and 45–50 years. The level of education was recoded into four categories: (1) *have not completed upper secondary school*, (2) *upper secondary school*, (3) *higher education, lower level (bachelor’s degree)*, and (4) *higher education, higher level (master’s degree or PhD)*. Relationship status was categorized as follows: (1) *in a relationship now*, (2) *was in a relationship before, but not now*, and (3) *have never been in a relationship*.

### Ethics

The study received approval from the Regional Committees for Medical and Health Research Ethics South East Norway (ref: 2021/295388), and the online survey used Nettskjema (https://nettskjema.no/?lang=en), a secure data collection tool for sensitive data developed by the University of Oslo. Participants were required to confirm that they had read the study information and agreed to participate by clicking the “I Agree” button. This action granted them access to the anonymous questionnaire. No incentive to participate in the study was offered. This article adheres to the STROBE guidelines for reporting cross-sectional research.

### Statistical analysis

Descriptive statistics (proportions for categorical variables, mean and standard deviations (*SD*) for continuous variables) were calculated. Chi-square tests were used to assess the difference in current menstruation aspects between the groups that used no or a nonhormonal type of contraception versus those that used hormonal contraception. One-way analysis of variance (ANOVA) tested the mean difference in menstrual attitudes between the four contraceptive use groups. Partial eta-squared (.01 (small), .06 (medium), and .14 (large)) was used to interpret the effect size of the group differences. A Bonferroni post hoc test was employed to test the difference between pairs of groups if a significant main effect was identified. A bivariate correlation analysis (Pearson’s *r*) was performed to examine the relationships between the study variables. To assess the effect size of the correlations, the guidelines of Cohen^39^ were used: *r* = .10 (small), *r* = .30 (medium), and *r* > .50 (strong). We conducted three hierarchical linear regression analyses to examine the unique contribution of the independent variables to the prediction of each of the three menstrual attitudes. Only independent variables that were significantly correlated with one of the outcome variables were entered into the regression analyses. The effect sizes were estimated using 95% confidence intervals and *R*^2^. Due to the large sample size and number of tests, *p*-value less than .01 was used for the significance level. We ran the analyses with SPSS version 27.

## Results

Over half of the 1470 participants fell within the age range of 25–34 years (Table 1), with the largest subgroup being 30–34 years old. The majority of participants had either completed or were in the process of completing higher education, while small minority had not finished upper secondary school. Most participants reported being in a relationship. The average age of menarche was 12.6 years, ranging from 8 to 18 years, and 42% indicated that they had little or no knowledge about menstruation at that time of their first period. In addition, 44.7% of the sample reported using hormonal contraception, including both long-acting and short-term alternatives. Among all participants, 13.4% reported using hormone-free contraception, while 41.5% stated that they did not use any form of contraception.

**Table 1.** Distribution of variables on sociodemographic background, contraceptive method, and age and knowledge at menarche (percentage).

	*N*	%
Age (years)		
18–24	174	11.8
25–29	390	26.5
30–34	430	29.3
35–39	250	17.0
40–44	129	8.8
45–50	97	6.6
Education		
Not completed upper secondary school	92	6.3
Upper secondary school	323	22.0
Higher education, lower level	493	33.6
Higher education, higher level	561	38.2
Relationship status		
In a relationship	1114	76.7
In a relationship before, but not now	283	19.5
Never been in a relationship	55	3.8
Contraceptive method		
Long-term hormonal contraception	323	22.1
Other hormonal contraception	333	22.8
Hormone-free contraception	195	13.4
Do not use contraception	609	41.7
Menarche (first menstruation)		
*Age (years)*		
⩽10	85	6.6
11	208	16.0
12	368	28.4
13	300	23.1
14	184	14.2
⩾15	151	11.7
*Knowledge*		
None	142	9.7
Very little	475	32.3
Some	594	40.4
A lot	217	14.8
Very much	41	2.8

Table 2 illustrates the differences in the distribution of variables related to current menstruation across contraception groups. The majority of participants reported regular monthly menstruation, with over half experiencing a period duration of 4–5 days, and half reporting moderate menstrual pain. Approximately, one-third reported experiencing much or very much pain during menstruation. The group using hormonal contraceptives reported significantly more irregular and less frequent menstrual periods, shorter periods, and less bleeding compared to the group using either nonhormonal or no contraceptives. There was no difference regarding the prevalence of menstrual pain between the two contraceptive groups.

**Table 2.** Difference in the distribution of variables related to current menstruation between contraception groups (chi-square).

	Total		Hormonal contraception		Nonhormonal/ no contraception		χ^2^
	*N*	%	*n*	%	*n*	%	
Current menstruation							
Frequency of periods							
Twice or more per month	62	4.3	41	6.3	21	2.6	
Regularly once a month	843	58.1	207	31.8	636	79.6	
Regularly/irregularly every 2/3 months	263	18.2	180	27.6	83	10.4	
Once or twice a year	115	7.9	98	15.1	17	2.1	
Never	169	11.5	125	19.2	42	5.3	347.4**
Period length (days)							
1–3	224	17.1	134	25.1	90	11.6	
4–5	742	56.6	246	46.1	496	63.8	
>6	345	26.3	154	28.8	191	24.6	53.64**
Amount of menstrual bleeding							
Nothing/a little	74	5.7	66	12.3	8	1.0	
Medium	865	65.9	361	67.5	504	64.9	
A lot/very much	373	25.4	108	20.2	265	34.1	93.74**
Menstrual pain							
Nothing/a little	221	16.8	107	20.0	114	14.7	
Medium	681	51.9	270	50.4	411	53.0	
A lot/very much	410	31.3	159	29.6	251	32.3	6.37

Note: ** = *p* < .001; χ^2^ = chi-square test.

The ANOVA tests (Table 3) revealed significant differences in women’s menstrual attitudes in relation to the type of contraception they used. Women who used nonhormonal contraceptives or no contraceptives at all agreed more with the attitude that menstruation is something natural, and both groups using hormonal contraception scored higher than nonusers on the attitude of menstruation as something bothersome. All significant group differences had small effect sizes.

**Table 3.** Differences in menstrual attitudes between different types of contraceptive methods (ANOVA).

Contraceptive method	Long-term hormonal (A)		Other hormonal (B)		Hormone-free (C)		Do not use (D)		*F*	*p*	η^2^	Post hoc
Menstrual attitude	*n*	*M* (*SD*)	*n*	*M* (*SD*)	*n*	*M* (*SD*)	*n*	*M* (*SD*)				
Natural	323	4.21 (1.22)	333	4.16 (1.18)	195	4.61 (1.29)	609	4.55 (1.29)	11.39	<.001	.02	C, D > A, B
Shameful	323	3.51 (1.22)	333	3.60 (1.24)	195	3.28 (1.19)	609	3.46 (1.27)	2.75	.042	.006	-
Bothersome	323	5.54 (1.34)	333	5.76 (1.13)	195	5.33 (1.47)	609	5.20 (1.38)	14.24	<.001	.03	A > D; B > C, D

Note: η^2^ = Partial eta-squared (effect size: 0.01 = small; 0.06 = medium; 0.14 = large); Post hoc = Bonferroni post hoc test.

Descriptive statistics and the bivariate correlations between the study variables are presented in Table 4. The mean scores on the menstrual attitude subscale indicated that the women on average considered menstruation to be more bothersome than shameful. Those who experienced menstruation as a natural part of being a woman also tended to consider it less bothersome or shameful. Agreeing with the attitude of menstruation as something natural correlated with reporting more positive emotions at first menstruation, the current menstruation being less painful, a more positive genital self-image, and experiencing fewer perimenstrual psychological symptoms. Menstruation as something bothersome was associated with experiencing more menstrual pain, being younger, having lower education level, negative emotions at menarche, more perimenstrual psychological symptoms, shorter period length, and negative genital self-image. The attitude of menstruation as something shameful was associated with lower sexual openness and negative genital self-image, with lower education level, and more negative emotions, fewer positive emotions, and less knowledge about menstruation at menarche. All the correlations with menstrual attitudes were weak or had a medium effect size. Among the independent variables, there was a strong correlation between more sexual openness and positive genital self-image.

**Table 4.** Correlations (Pearson’s *r*) between study variables, mean, and standard deviation.

	MA-N	MA-S	MA-B	AgeM	KnM	Emo-P	Emo-N	Length	Bleed	Pain	Symp	SxOp	GenS	Edu	*n*	*M*	*SD*	Min–Max
MA natural	-														1470	4.40	1.26	1–7
MA shameful	−.22**	-													1470	3.48	1.25	1–7
MA bothersome	−.44**	.23**	-												1470	5.42	1.34	1–7
Age menarche	.06	−.00	−.10**	-											1296	12.6	1.57	8–18
Knowledge men	.01	−.17**	−.05	.23**	-										1469	2.69	0.93	1–5
Emo-Pos men	.17**	−.15**	−.08*	.24**	.35**	-									1468	2.74	1.18	1–5
Emo-Neg men	−.04	.23**	.12**	−.25***	−.43**	−.53**	-								1468	2.92	1.10	1–5
Period length	−.10**	.05	.12**	−.09*	−.02	−.02	.09**	-							1311	2.09	0.65	1–3
Period bleeding	−.09*	.06	−.10**	−.10**	−.08*	.00	.07*	.48**	-						1312	4.77	1.37	1–7
Period. pain	−.20**	.05	.22**	−.16**	−.09*	−.02	.13**	.24**	.47**	-					1311	4.43	1.78	1–7
Period symp	−.12**	.11**	.16**	−.07	−.06	.08*	.20**	.10**	.16**	.40**					1302	3.37	1.08	1–5
Sexual openness	.11*	−.44**	.02	−.01	.15**	.17**	−.16**	−.03	.01	.00	−.03	-			1469	5.22	0.96	1.4–7
Genital self-image	.24**	−.41**	−.12**	.02	.18**	.14**	−.23**	−.09*	−.08*	−.16**	−.19**	.50**	-		1469	3.01	0.58	1–4
Education	.07*	−.14**	−.14**	.11**	.15**	.10**	−.10**	−.10**	−.17**	−.22**	−.10**	.08*	.19**	-	1469	4.87	1.79	1–7
Age	.00	.01	−.12**	.06	.01	.00	−.06	−.04	.01	−.14**	−.12**	−.04	.08*	.06	1470	3.04	1.36	1–6

Note: MA = Menstrual attitude; MA-N = Menstrual attitude—Natural; MA-S = Menstrual attitude—Shameful; MA-B = Menstrual attitude—Bothersome; AgeM = Age at menarche; Knowledge Men/KnM = Knowledge at menarche; Emo-Pos Men/Emo-P = Positive emotions at menarche; Emo-Neg Men/Emo-N = Negative emotions at menarche; Length = Period length; Bleed = Menstrual bleeding; Pain = Menstrual pain; Period Symp/Symp = Perimenstrual psychological symptoms; SxOp = Sexual openness; GenS = Genital self-image; Edu = Educational level.

* = *p* < .01; ** = *p* < .001.

Table 5 displays the outcomes from hierarchical multiple linear regression analyses for each of the three distinct menstrual attitudes as dependent variables. The independent variables included in the models remained consistent across each outcome variable. The first model incorporated age and education level alongside variables pertaining to menarche. In the second step, variables related to current menstruation and contraceptive use were introduced in the model, while the third model included the sexual openness and genital self-image variables. Each regression analysis is identified with a heading specifying the respective outcome variable. Due to the significant impact of hormonal contraceptive use on menstrual cycles, the variable related to contraceptive use was included in the analysis in lieu of the variables concerning period length and menstrual bleeding.

**Table 5.** Hierarchical multiple linear regression analysis with menstrual attitudes as the criterion.

Menstrual attitudes	As natural *n* = 1147			As shameful *n* = 1147			As bothersome *n* = 1147		
	β	*p*	CI 95%	β	*p*	CI 95%	β	*p*	CI 95%
Model 1 *R*^2^	.**038**			.**063**			.**052**		
Age	−.003	.92	−.06, .05	−.01	.67	−.06, .04	**−.11**	**<.001**	**−.17, −.06**
Education	.**08**	.**006**	.**02, .10**	**−.09**	.**002**	**−.10, −.02**	**−.13**	**<.001**	**−.14, −.05**
*Menarche*									
Age first period	.04	.22	−.02, .08	.07	.023	.01, .10	−.05	.07	−.10, .01
Knowledge	−.06	.05	−.17, .00	−.08	.014	−.19, −.02	.04	.21	−.03, .15
Emotions: proud/happy	.**19**	**<.001**	.**13, .27**	.005	.89	−.06, .08	−.02	.64	−.10, .06
Emotions: concerned/afraid	.04	.24	−.03, .13	.**19**	**<.001**	.**13, .29**	.**11**	.**003**	.**04, .22**
Model 2 Δ *R*^2^	.**068**			.**007**			.**058**		
Age	−.07	.016	−.12, −.01	.01	.85	−.05, .06	−.04	.15	−.10, .02
Education	.03	.33	−.02, .06	−.09	.**004**	**−.10, −.02**	**−.08**	.**006**	**−.11, −.02**
*Menarche*									
Age first period	.01	.86	−.04, .05	.07	.02	.01, .10	−.02	.40	−.07, .03
Knowledge	−.06	.08	−.16, .01	−.09	.010	−.20, −.03	.03	.30	−.04, .14
Emotions: proud/happy	.**23**	**<.001**	.**17, .31**	−.01	.76	−.08, .06	−.05	.14	−.13, .02
Emotions: concerned/afraid	.**10**	.**006**	.**03, .19**	.**17**	**<.001**	.**10, .26**	.06	.11	−.02, .16
*Current menstruation*									
Psychological symptoms	**−.10**	.**001**	**−.19, −.05**	.08	.02	.01, .16	.**09**	.**004**	.**04, .19**
Pain	**−.19**	**<.001**	**−.17, −.09**	−.02	.56	−.06, .03	.**16**	**<.001**	.**08, .17**
No/nonhormonal (0) versus hormonal contraception (1)	**−.16**	**<.001**	**−.54, −.25**	.05	.07	−.01, .28	.**17**	**<.001**	.**29, .61**
Model 3 Δ *R*^2^	.**038**			.**196**			.**005**		
Age	**−.08**	.**006**	**−.13, −.02**	.01	.80	−.04, .06	−.04	.19	−.10, .02
Education	.003	.92	−.04, .04	−.04	.13	−.06, .01	−.08	.010	−.11, .01
*Menarche*									
Age first period	.02	.41	−.03, .06	.02	.42	−.02, .06	−.03	38	−.07, .03
Knowledge	−.08	.02	−.18, −.02	−.05	.12	−.14, −.02	.04	.26	−.04, .14
Emotions: proud/happy	.**22**	**<.001**	.**16, .30**	.04	.17	−.02, .11	−.05	.12	−.14, .02
Emotions: concerned/afraid	.**12**	**<.001**	.**06, .22**	.**11**	**<.001**	.**05, .19**	.05	.14	−.02, .15
*Current menstruation*									
Psychological symptoms	−.08	.014	−.16, −.02	.04	.16	−.02, .11	.**08**	.**009**	.**03, .18**
Pain	**−.17**	**<.001**	**−.16, −.08**	−.03	.32	−.06, .02	.**16**	**<.001**	.**07, .16**
No/nonhormonal (0) versus hormonal contraception (1)	**−.15**	**<.001**	**−.52, −.24**	.06	.03	.01, .27	.**16**	**<.001**	.**28, .60**
Sexual openness	−.03	.29	−.12, .04	**−.31**	**<.001**	**−.48, −.33**	.07	.04	.01, .18
Genital self-image	.**22**	**<.001**	.**33, .61**	**−.22**	**<.001**	**−.59, −.33**	−.08	.02	−.33, −.02
Total *R*^2^	.**136**			.**266**			.**114**		

### Menstruation as something natural

Higher education and positive emotions at the time of menarche predicted the attitude of menstruation as a natural part of being a woman, with only 3.8% of the variance explained in the first model. All variables related to current menstruation that were added in the second model were significant predictors (Δ *R*^2^ = 6.8%). The explained variance increased by an additional 3.8% to a total of 14.4% when the psychological variables were included in the third model. Less menstrual pain, use of no or hormone-free contraception, both more positive and negative emotions at menarche, and a positive genital self-image were all significant predictors in the attitude of menstruation as something natural in the final model.

### Menstruation as something shameful

Lower education level and negative emotions at menarche were the only significant predictors of the attitude of menstruation as something shameful in the first model, with an explained variance of 6.3%. Less knowledge at menarche also emerged as a significant predictor in the second model, while none of the variables related to current menstruation were significant (Δ *R*^2^ = .7%). However, when the variables sexual openness and genital self-image were included, the explained variance increased by 19.6% to a total of 26.6% for the final model. Less sexual openness and negative genital self-image, and to a lesser degree, more negative emotions at first menstruation, were the final significant variables predicting the attitude of menstruation as something shameful.

### Menstruation as something bothersome

In predicting the attitude of menstruation as something bothersome, the first model explained 5.2% of the variance. In the second model (Δ *R*^2^ = 5.8%), more menstrual pain and hormonal contraception were the strongest significant predictors. Adding sexual openness and genital self-image in the final model only increased the explained variance by 0.5% (with a total of 11.5%) and did not change the significant predictors from the previous model.

## Discussion

To our knowledge, this is the first study to examine Norwegian women’s menstrual attitudes. The overall aim of the study was to investigate adult women’s attitudes toward menstruation and to study how various factors linked to sociodemographic background, experience of first and current menstruation, contraceptive method, sexual openness, and genital self-image were associated with and predicted menstrual attitudes. The results showed that menstrual attitudes in Norwegian women largely corresponded with the three separate dimensions previously described in the literature: menstruation as something natural, shameful, and bothersome.^14,37^

Menstruation as something natural was predicted more by positive than negative emotions at menarche, by less current menstrual pain, using nonhormonal or no contraception, and by a more positive genital self-image. In addition to lower educational level, perceiving menstruation as bothersome was only predicted by variables related to current menstruation, such as stronger menstrual pain, more perimenstrual psychological symptoms, and using hormonal contraceptives. Menstruation as something shameful differed from the other two attitudes by not being predicted by current menstruation factors but by the strong association with lower sexual openness and a negative genital self-image and to some degree with negative emotions at menarche.

### Multidimensional and ambivalent menstrual attitudes

The finding that the women tended to express attitudes toward menstruation as both natural and bothersome supports the idea of menstrual attitudes as a multidimensional concept^37^ and corresponds with the findings from previous research.^3,18^ Attitudinal ambivalence encompasses the coexistence of both positive and negative evaluations simultaneously.^40^ For instance, women may perceive menstruation as a natural process while also finding it bothersome in their daily lives. Such ambivalent attitudes can stem from conflicts between core values, beliefs, and behaviors. This conflict may arise from exposure to inconsistent information and external attitudes toward menstruation.^40^ Contradictory social narratives regarding menstruation, portraying it as both an obstacle to women’s lives and health, or as unnecessary and useless, coexist alongside perspectives that regard monthly menstruation as a sign of health^41,42^ and questions the health consequences of using hormonal contraception.^43,44^ Furthermore, the social stigma associated with menstrual blood can lead women who initially had positive thoughts and feelings related to their own menstruation and considered it a natural part of being a woman to subject themselves to social restrictions associated with secrecy and concealment to avoid being judged and treated negatively by others.

### Sociodemographic background and menarche

The correlational analysis revealed a relatively small bivariate relationship between lower education level and attitudes toward menstruation as both something shameful and bothersome. Education remained a significant, albeit weak, predictor for menstruation as bothersome in the final regression model. According to the health gradient, women with lower education have less access to healthcare and information,^45^ which is consistent with research that has found that having less or incorrect information about menstruation increases the likelihood that women experience menstruation as physically and psychologically debilitating.^24^ In addition, health information and good access to health services may alleviate menstruation-related problems and thus lead to experiencing menstruation as less of a burden.

Experience of menarche has been found to lay a decisive foundation for later menstrual attitudes in women.^23,26,46^ Over 40% of the women in our study reported that they had no or very little knowledge when they got their first period, which in turn had a small bivariate association with the attitude toward menstruation as something shameful. The information that is available at the time of menarche is influenced by external factors, such as media, parents, and peers, and menstrual attitude formation will thus be susceptible to social discourses of menstruation as shameful and “dirty.”^25^ For example, we found that both younger age and less knowledge at menarche were correlated with feelings of worry, shame, and fear, while being older and having more knowledge at first menstruation were more associated with feelings of pride and joy. In line with this, several studies have found that girls who get their first period early compared to their peers often have a more negative experience at the time and more negative attitudes later in life.^23,46,47^ The relationship between age and emotional reaction at menarche may be viewed in a sociocultural context where menarche is a sign of reproductive potential and sexual maturity, even though girls on average get their first period several years before their sexual debut and almost two decades before their first pregnancy.^48^ Schooler et al.^15^ problematized how young girls often learn about menstruation in connection with sex education at school, which may lead to a more sexualized perspective on menstruation.

Our findings emphasize the lasting impact of emotional responses to menarche on the formation and consistency of menstrual attitudes. It is logical that the negative emotions experienced at menarche predicted both viewing menstruation as something shameful and as bothersome. However, the finding that both positive and negative emotions at menarche predicted the adult attitude of menstruation as something natural is interesting. One interpretation could be that this reflects the complex and contradictory societal messages surrounding teenage girls’ first menstruation—viewed as both a source of pride and shame. Consequently, these initial contrasting perceptions of menstruation may have been assimilated into the perception of its natural aspects, encompassing both positive and negative attributes.

### Current menstruation and contraceptive use

Our results showed that associations between menstrual attitudes and physiological factors were linked to current menstruation. In the correlation analysis, period length, amount of bleeding, perimenstrual psychological problems, and menstrual pain were all negatively associated with the attitude about menstruation as something natural and positively associated with regarding menstruation as bothersome. This is consistent with previous research that found that women who experienced irregular periods, heavy bleeding, and menstrual pain reported more negative attitudes toward menstruation than women with more regular, lighter, and less painful periods.^49,50^ It makes sense that women with a menstruation that negatively impacts their daily life were more likely to agree with scale items, such as that men have an advantage in not having periods or hoping one day it will be possible to have periods for just a few minutes.

Our findings indicate that an important aspect of the use of hormonal contraception is to regulate menstruation, especially to obtain fewer and shorter periods. Despite this association, there is still little research into how contraception may relate to menstrual attitudes. To date, studies have found that negative attitudes toward menstruation, or attitudes toward menstruation as something troublesome or less natural, were associated with positive attitudes toward menstrual suppression through the use of hormonal contraception.^29,30,51^

One contribution from the current study was to test the differences in menstrual attitudes between contraceptive methods in more detail and to explore the extent to which contraceptive use predicted the various attitude dimensions when the other variables were held constant. The results showed that women who used no or hormone-free contraception on average viewed menstruation as something natural, while women who used hormonal contraceptive methods were more likely to have the attitude toward menstruation as something bothersome. These associations remained significant in the regression analysis when other relevant predictors were controlled for, illustrating the importance of taking different types of contraceptive use into consideration when studying menstrual attitudes. However, this does not imply a causal direction, as women may have many different reasons for using hormonal, hormone-free, or no contraception. It is plausible that women who experience menstruation as something bothersome are more likely to use hormonal contraception to control their periods and make them less disruptive in everyday life. However, using hormonal contraception may reinforce their view that menstruation is mostly negative for women. It is conceivable that women who find menstruation to be a natural part of being a woman will be more comfortable with using contraceptive methods that allow periods to occur naturally. Their tendency to prefer hormone-free or no contraception may also be related to experiencing less, or having higher tolerance for, negative symptoms when menstruating.

The finding that 41.7% of the women reported not using any form of contraception is somewhat higher than the 37.6% found in a nationwide Swedish study.^52^ The difference may be explained by the fact that the majority of women in the current study were between 25 and 34 years old, which is an age range when many are trying to become pregnant or have given birth.^53^

### Menstrual shame, sexual openness, and genital self-image

The results from this study showed that both negative genital self-image and less sexual openness were more strongly associated with the attitude toward menstruation as something shameful than with the other two attitude dimensions. This suggests that perceiving menstruation as shameful may be qualitatively distinct from regarding it as natural or bothersome. While a positive genital self-image, but not sexual openness, predicted the attitude of menstruation as natural, it is plausible that considering menstruation as natural or bothersome is more closely associated with factors directly related to menstruation itself. Conversely, menstrual shame may predominantly reflect societal and sexualized aspects tied to self-objectification, reproductive functioning, and taboo. One interesting path to pursue in future research is the role of self-objectified body surveillance in how thoughts and feelings related to menstruation, sexuality, and the genitals are interconnected and developed in a triad of shame.

There was also a strong association between genital self-image and sexual openness, which may be equated with findings from Komarnicky et al.^54^ showing that a positive genital self-image was associated with both higher sexual functioning and sexual satisfaction in women. However, genital self-image and sexual openness were also both unique predictors for the attitude of menstruation as something shameful in the multiple regression analysis. That genital self-image which is an important aspect of women’s menstrual attitudes in itself is supported by similar findings from qualitative studies on women’s thoughts and feelings toward their own genital area, where menstruation-related themes were regularly linked to disgust and contempt, and fear of smell.^34–36^ While negative thoughts and feelings about the genital area may be transferred onto attitudes about menstruation, a reverse causal direction is also possible: shame related to menstruation can transfer to body shame of the genital area and even to the body in general.^15^

Considering menstruation shameful was also predicted by less sexual openness both publicly and in private. This is consistent with research that has found that being comfortable with one’s sexuality is linked to viewing menstruation as normal and acceptable.^13^

### Strengths and limitations

The main strength of this study was the examination of menstrual attitudes among a relatively large sample of adult Norwegian women, which, to our knowledge, has not been done previously. The study also included rarely studied aspects of menstrual attitudes, such as contraceptive use, sexual openness, and genital self-image. The study has several limitations that need to be considered as well. The project title “Menstruation and health” may have caused individuals with negative attitudes toward menstruation to decline participation in the study, potentially resulting in a bias toward positive attitudes in the obtained results. In addition, the sample had a preponderance of highly educated Norwegian women. On a national basis, approximately 40% of Norwegian women have higher education,^55^ while the proportion in this study was more than 70%. It is therefore important to interpret the findings from the study in light of this sampling bias. Based on our findings, it appears that women with higher education tended to agree less with the attitudes that menstruation is shameful and bothersome. It is thus possible that this study underestimated the degree of agreement with these attitudinal dimensions among Norwegian women.

Another limitation relates to the validity of the scale used to measure menstrual attitudes. The factor analysis that was carried out in our sample showed a slightly different factor solution than in similar studies from the United States. In this study, for example, the statement “Menstruation is something I have to put up with” loaded on the attitude toward menstruation as something natural, while it loaded on the attitude toward menstruation as something bothersome in American studies.^14,38^ This could indicate that Norwegian women interpreted the statements differently or that the results reflect real, but small, cultural differences in menstrual attitudes.

### Practical implications and further research

The results of this study support previous research that has found that menstrual attitudes are linked to several aspects of women’s lives and health, including sexuality and body image. Schooler et al.^15^ explored the connection between menstrual shame, body shame, and sexual behavior and decision-making, and future research should explore these interconnected topics more closely, particularly in relation to genital self-image and sexual openness and the role menstrual shame plays in a sexual context. Future research should also explore women’s perceptions, feelings, and satisfaction related to contraceptive methods, as this is closely linked to menstrual attitudes and sexual health. Findings from our study have also emphasized the importance of preparing girls for their first menstruation in preventive health work and focusing not only on knowledge but also on expectations and emotions.

As the work by Schmalenberger et al.^56^ pointed out, further studies should also develop consistent methods for the operationalization of menstruation and the menstrual cycle so that research results can be more easily compared. This applies, for example, to whether the participants have a natural cycle or not but also to whether all types of bleeding from the vagina should be defined as menstruation, as some contraceptives may lead to bleeding in-between periods. Many studies also simplify the discussion of menstrual attitudes by referring to these as positive or negative, even though attitudes seem to be complex and multidimensional.

## Conclusion

This study showed that Norwegian women’s attitudes toward menstruation are multidimensional and ambivalent, with some having attitudes about menstruation as both natural and bothersome. The attitude about menstruation as shameful differed in the pattern of predictors from the other two attitudinal dimensions, which may be due to the social stigma associated with menstruation. Emotional experience at first menstruation, contraceptive method, sexual openness, and genital self-image emerged as central factors in predicting menstrual attitudes in adult women. More research about how menstruation relates to contraception use, sexuality, and body image is needed to expand the existing knowledge based on the furtherance of women’s menstrual health.

## Supplemental Material

sj-docx-1-whe-10.1177_17455057241249553 – Supplemental material for Menstrual attitudes in adult women: A cross-sectional study on the association with menstruation factors, contraceptive use, genital self-image, and sexual openness Supplemental material, sj-docx-1-whe-10.1177_17455057241249553 for Menstrual attitudes in adult women: A cross-sectional study on the association with menstruation factors, contraceptive use, genital self-image, and sexual openness by Ingela Lundin Kvalem, Ida Maria Dahr Nygaard, Bente Træen, Anna Ivanova and Camilla Lindvall Dahlgren in Women’s Health

sj-docx-2-whe-10.1177_17455057241249553 – Supplemental material for Menstrual attitudes in adult women: A cross-sectional study on the association with menstruation factors, contraceptive use, genital self-image, and sexual openness Supplemental material, sj-docx-2-whe-10.1177_17455057241249553 for Menstrual attitudes in adult women: A cross-sectional study on the association with menstruation factors, contraceptive use, genital self-image, and sexual openness by Ingela Lundin Kvalem, Ida Maria Dahr Nygaard, Bente Træen, Anna Ivanova and Camilla Lindvall Dahlgren in Women’s Health

## Declarations

**Ethics approval and consent to participate:** The study was approved by the Regional Committees for Medical Research Ethics South East Norway, REK South East (ref: 2021/295388), and the online survey used a secure data collection tool for sensitive data. Consent to participate in the study was given by clicking on the “I agree” button which gave access to the anonymous questionnaire.

**Consent for publication:** Not applicable.

**Availability of data and materials:** The questionnaire used in the study has been included in the Supplemental material. Data are not available due to ethical and legal restrictions
