# Supplementary material for: Menstrual attitudes in adult women: A cross-sectional study on the association with menstruation factors, contraceptive use, genital self-image, and sexual openness
Source: Womens Health (Lond). 2024 Apr 29;20:17455057241249553. doi: 10.1177/17455057241249553 (PMC11060024; doi:10.1177/17455057241249553)
Supplement: sj-docx-1-whe-10.1177_17455057241249553 – Supplemental material for Menstrual attitudes in adult women: A cross-sectional study on the association with menstruation factors, contraceptive use, genital self-image, and sexual openness [file sj-docx-1-whe-10.1177_17455057241249553.docx]

**Supplement Table**

*Factor Analysis Solution of Menstrual Attitudes (n = 1470)*

|  | Rotated factor solutions | | |
| --- | --- | --- | --- |
|  | 1 | 2 | 3 |
| **Factor 1: Menstruation as something natural** | | | |
| 13. Menstruation is a reoccurring affirmation of womanhood | **.80** | -.03 | -.13 |
| 15. Menstruation is an obvious example of the rhythmicity which pervades all of life | **.80** | -.07 | -.13 |
| 14. Menstruation allows women to be more aware of their bodies | **.79** | -.10 | -.18 |
| 16. The recurrent monthly flow of menstruation is an external indication of a woman’s general good health | **.70** | .03 | -.07 |
| 12. Menstruation provides a way for me to keep in touch with my body | **.68** | -.22 | -.36 |
| 2. In some ways I enjoy my menstrual periods. | **.55** | -.14 | -.47 |
| 1. Menstruation is something I have to put up with | **.48** | -.04 | .03 |
| **Factor 2: Menstruation as something shameful** |  |  |  |
| 9. I would prefer not to talk openly about menstruation | -.07 | **.79** | -.08 |
| 11. When I have my period, I do things to hide the fact that I am menstruating | -.01 | **.78** | -.08 |
| 8. I am embarrassed when I have to purchase menstrual products | .00 | **.70** | -.07 |
| 10. I find menstrual blood disgusting | -.14 | **.67** | .24 |
| 6. I would feel ashamed if I “leaked” menstrual blood on my clothes | -.09 | **.62** | .08 |
| 7. I avoid touching my genital region when I am menstruating | -.01 | **.54** | .30 |
| **Factor 3: Menstruation as something bothersome** |  |  |  |
| 4. I hope it will be possible someday to get a menstrual period over within a few minutes | -.23 | .07 | **.77** |
| 3. Men have a real advantage in not having the monthly interruption of a menstrual period | -.08 | .05 | **.75** |
| 5. The only thing menstruation is good for is to let me know I’m not pregnant | -.12 | .11 | **.74** |
| Eigenvalue | 4.71 | 2.60 | 1.46 |
| % Explained variance | 29.41 | 16.24 | 9.14 |
| Total explained variance | 54.80 | | |
