# Supplementary material for: Menstrual attitudes in adult women: A cross-sectional study on the association with menstruation factors, contraceptive use, genital self-image, and sexual openness
Source: Womens Health (Lond). 2024 Apr 29;20:17455057241249553. doi: 10.1177/17455057241249553 (PMC11060024; doi:10.1177/17455057241249553)
Supplement: sj-docx-2-whe-10.1177_17455057241249553 – Supplemental material for Menstrual attitudes in adult women: A cross-sectional study on the association with menstruation factors, contraceptive use, genital self-image, and sexual openness [file sj-docx-2-whe-10.1177_17455057241249553.docx]

**Study: “Menstrual attitudes in adult women” Questions used in the online survey**

**(web-based tool: “Nettskjema”)**

| **Q** | **Question** | **Response categories** |
| --- | --- | --- |
| Inclusion questions | **Are you?** | Female  Male  Other  Between 18 and 50 years of age |
|  | **SOCIO-DEMOGRAPHIC VARIABLES** | |
| 1 | **Age** | 18-24 år  25-29 år  30-34 år  35-39 år  40-44 år  45-50 år |
| 2 | **What is your sexual orientation?** | Heterosexual  Lesbian/Homosexual  Bisexual or pansexual  Asexual  Other  Do not know  Prefer not to answer |
| 3 | **What is the highest educational level that you completed?** | Lower secondary school (9-10 years at school)  Higher secondary school, high school (12-13 years at school)  College, lower university level (Bachelor degree level or similar)  Higher university level (Master degree, Ph.D. level or similar) |
| 4 | **Apart from special occasions such as weddings, funerals and baptisms, how often do you attend services or meetings connected with your religion**? | Once a week or more  Once every two weeks  Once a month  Twice a year  Once a year  Less than once a year  Never |
| 5 | **Are you currently in a committed relationship?** | Yes  No  Other |
| 5_1 | If “No” on Q5 **How long has it been since your last steady relationship ended?** | Less than 1 year ago  1-3 years ago  3-5 years ago  More than 5 years ago  Have not been in a permanent relationship before |
| 5_2 | If “Yes” on Q5**How long have you been together?** | Less than 1 year  1-2 years  3-5 years  More than 5 years |

|  | **SEXUAL ACTIVITY AND SATISFACTION** | |
| --- | --- | --- |
| 6 | **Thinking about your sex life in the last year: All things considered, how satisfied are you with your sexual life?** | Completely dissatisfied  Dissatisfied  Neither dissatisfied nor satisfied  Satisfied  Completely satisfied |
| 7_1 to 7_6 | **During the last six months, how often on average have you participated in the following sexual activities:**  Masturbation (without partner present)  Vaginal intercourse  Oral sex (receive from partner, or give to partner)  Anal sex (stimulation of own or partner's anus with finger, penis, tongue or other)  Masturbation with a partner  Stimulation of erogenous zones other than the genitals | Never  Less often than once per month  1 to 3 times in the past month  Once a week  2 or 3 times a week  4 or 6 times a week  One or more than once a day |
| 8_1 to 8_10 | **Please answer the following questions as honestly as possible by ticking the answer that best suits your response:**  I feel comfortable talking about sexuality with strangers  I think I am a sexy person  I would feel comfortable explaining to a same sex sibling or friend how to masturbate  I wish everyone had an open attitude towards sexuality  Even when not in a relationship, I still feel like a sexual person  I do not feel comfortable watching movies or reading literature that I find sexually stimulating. [reversed]  I would not feel comfortable carefully examining my genitals. [reversed]  I am willing to act on new sexual ideas I get from others.  I use words like nipples, penis, vagina, or clitoris in conversation, with no trouble  The main purpose of sex is for people to enjoy themselves. | Strongly disagree  Moderately disagree  Mildly disagree  Neither agree nor disagree  Mildly agree  Moderately agree  Strongly agree |
| 9_1 to 9_7 | **Now we want to ask you some questions about your thoughts and feelings related to your genitalia.** Answer the questions below as honestly as possible by ticking the extent to which you agree or disagree with each statement  I feel positively about my genitals  I am satisfied with the appearance of my genitals  I would feel comfortable letting a sexual partner look at my genitals  I think my genitals smell fine  I think my genitals work the way they are supposed to work  I feel comfortable letting a healthcare provider examine my genitals  I am not embarrassed about my genitals | Strongly disagree  Disagree  Agree  Strongly agree |
|  | **MENSTRUATION** | |
| 10_1 to 10_16 | **Answer the questions below as honestly as possible by ticking the extent to which you agree or disagree with each statement**  Menstruation is something I have to put up with.  In some ways I enjoy my menstrual periods.  Men have a real advantage in not having the monthly interruption of a menstrual period.  I hope it will be possible someday to get a menstrual period over within a few minutes.  The only thing menstruation is good for is to let me know I’m not pregnant.  I would feel ashamed if I “leaked” menstrual blood on my clothes.  I avoid touching my genital region when I am menstruating.  I am embarrassed when I have to purchase menstrual products.  I would prefer not to talk openly about menstruation.  I find menstrual blood disgusting.  When I have my period, I do things to hide the fact that I am menstruating.  Menstruation provides a way for me to keep in touch with my body.  Menstruation is a reoccurring affirmation of womanhood.  Menstruation allows women to be more aware of their bodies.  Menstruation is an obvious example of the rhythmicity which pervades all of life.  The recurrent monthly flow of menstruation is an external indication of a woman’s general good health. | 1.Disagree strongly  2.  3.  4. Neither disagree, nor agree  5.  6.  7. Agree strongly |

| 11_1 to 11_8 | **When I think of my menstruation I feel:**  Anger  Disgust  Joy  Irritation  Sadness  Shame  Happiness  Pride | Not at all  To a small extent  Neither or  To some extent  Very much |
| --- | --- | --- |
|  | **CONTRACEPTIVE USE AND CURRENT MENSTRUATION** | |
| 12 | **Do you use any form of contraception?**  (If you use hormonal contraception in combination with a condom or other, indicate which hormonal contraception you use) | 1 Long-term hormonal contraception (contraceptive implant, hormonal intrauterine device (IUD))  2 Other hormonal contraception, (contraceptive pills, mini-pills, contraceptive ring/patch/injection)  3 Hormone-free contraception, (condom, diaphragm, nonhormonal IUD, fertility awareness methods (FAM), apps)  4 Do not use contraception  5 Other, please specify  6 Prefer not to answer |
| 13 | **On a scale from 0 to 6, where 0 is "very dissatisfied" and 6 is "very satisfied": how satisfied are you with the contraceptive method you use?** | Very dissatisfied  Quite dissatisfied  Slightly dissatisfied  Neither dissatisfied nor satisfied  Slightly satisfied  Quite satisfied  Very satisfied |
| 14 | **In what way(s) has the birth control you are on affected your period?** | Has not affected my period  Open answer, please specify below |
| 15 | **How often do you have your period/are you bleeding?**  (If you are on hormonal contraception that has changed or removed your period, please answer based on your current bleeding pattern) | Regularly once a month  Regularly, once every two or three months  Irregular, once to twice every three months  Irregular, once every six months  Irregular, less often than once a year  Never  Other, please specify  Prefer not to answer |
| 16 | **How many days do you usually bleed when you have your period?** | 1-3 days  4-5 days  6 days or more |
| 17/18 | **On a scale from 1 to 7, where 1 is “nothing” and 7 is “very much”, please answer the following:**  **How much do you tend to bleed when you have your period?**  **How much pain do you usually experience in connection with your menstruation?** | 1 Nothing  2  3  4  5  6  7 Very much |

| 19_1 to 19_8 | **To what extent do you currently experience other physical or psychological menstruation-related symptoms before or during your period?**  Bloating  Acne and/or pimples  Headaches/migraines  Low mood and/or sadness  Irritation  Anger and/or hostility  Increased sweet tooth  Anxiety | Not at all  To a small extent  Neither nor  To some extent  Very much |
| --- | --- | --- |
| 20 | **Have you been diagnosed with, or suspect you have, a menstrual-related illness?** | No  Yes, please specify |
|  | **MENARCHE** | |
| 21 | **About how old were you when you first got your period?** | Open answer |
| 22 | **When did you get your first period compared to your peers?** | Earlier than others  About the same time as others  Later than others |
| 23 | **How much knowledge did you have about menstruation when you got your period for the first time?** | No knowledge  Very little knowledge  Some knowledge  Quite a lot of knowledge  Very much knowledge |
| 24_1 to 24_9 | **To what extent did you experience the emotions mentioned below when you had your first menstruation?**  Excited  Shameful  Mature  Afraid  Happy  Sad  Worried  Weird  Proud | To a very small extent  To a small extent  Neither or  To some extent  To a very large extent |
| 25 | **How well prepared were you when you got your period for the first time?** | Very little prepared  Little prepared  Somewhat prepared  Very well prepared |
|  | **Thank you for answering this survey! We really appreciate you taking the time to share your experiences with us.**  **If you have any questions, comments, express your own thoughts and/or concerns about the topics, or feel the need to talk to someone after this survey, you can contact Ingela Lundin Kvalem** | |
